# Targeting the Mutational Landscape of Bystander Cells: Drug-Promoted Blood Cancer From High-Prevalence Pre-neoplasias in Patients on BRAF Inhibitors

**DOI:** 10.3389/fonc.2020.540030

**Published:** 2020-09-11

**Authors:** Donjete Simnica, Harald Ittrich, Carsten Bockemeyer, Alexander Stein, Mascha Binder

**Affiliations:** ^1^Department of Internal Medicine IV, Oncology/Hematology, Martin-Luther-University Halle-Wittenberg, Halle (Saale), Germany; ^2^Department of Oncology and Hematology, BMT with section Pneumology, Hubertus Wald Tumorzentrum/UCCH, University Medical Center Hamburg-Eppendorf, Hamburg, Germany; ^3^Department of Diagnostic and Interventional Radiology and Nuclear Medicine, University Medical Center Hamburg-Eppendorf, Hamburg, Germany; ^4^Hematology-Oncology Practice Hamburg (HOPE), Hamburg, Germany

**Keywords:** chronic lymphocytic leukemia, monoclonal B cell lymphocytosis, BRAF inhibition, hairy cell leukemia, RAS mutation

## Abstract

Drug-promoted cancers are increasingly recognized as a serious clinical problem in patients receiving BRAF inhibitory treatment. Here we report on a patient with *BRAF* mutant hairy cell leukemia and monoclonal B-cell lymphocytosis (MBL), who responded durably to BRAF/MEK inhibitors (BRAFi/MEKi) but experienced transformation of a *RAS* mutant MBL to chronic lymphocytic leukemia (CLL) with accelerated nodal progression. Hypothesizing that BRAFi triggered excessive MEK-ERK signaling in the MBL/CLL clone via the CRAF/RAS complex as previously described for BRAFi-induced cancers, BRAFi was discontinued inducing a rapid remission of the CLL on MEKi alone. Liquid biopsy monitoring showed a continuous increase of the MBL/CLL clone from the start of BRAFi/MEKi treatment followed by a rapid decline upon BRAFi withdrawal. Next-generation sequencing of a cohort of patients with MBL and monoclonal gammopathy of unclear significance (MGUS) revealed that almost one third of these cases harbored *RAS* mutations. In view of the population frequency of lymphatic pre-malignant conditions and the prevalence of *RAS* mutations in such cases, vigilant surveillance remains critical in patients treated with BRAF inhibitors.

## Introduction

Activating *BRAF* mutations (most prominently *BRAF* V600E), providing oncogenic signaling through the mitogen-activated protein kinase (MAPK) pathway, are the key molecular driver in a variety of solid tumors including roughly 50% of malignant melanomas, 15% of thyroid cancers, 8% of colorectal cancers, 3% of lung cancers, and 2% of pancreatic cancers. In hematological malignancies, *BRAF* mutations occur in approximately 7% of multiple myeloma and almost 100% of classical hairy cell leukemias (HCL) (1). Small molecule BRAF inhibitors (BRAFi) are increasingly used for therapeutic targeting with and without concomitant MEK inhibitors (MEKi) yielding convincing clinical results.

Despite the fact that BRAF is a key effector downstream of RAS and upstream of MEK, ATP-competitive BRAFi are not effective in *RAS* mutant tumor models (2). In fact, such inhibitors have opposing effects on MAPK signaling depending on the *BRAF* mutational status. In *BRAF* V600E mutant tumors, BRAFi effectively block MAPK signaling and inhibit tumor growth. However, in tumor and normal cells expressing wild-type BRAF, these inhibitors have been found to promote BRAF dimerization, activation and binding to RAS-GTP, ultimately resulting in stimulation of MEK-ERK signaling and proliferative effects (3, 4). This paradoxical activation is thought to explain why this class of drugs may induce or promote *RAS* mutant neoplasias which emerges as a more and more serious clinical problem the more patients are offered this targeted treatment approach (5–12).

Here we report on a patient treated with BRAFi/MEKi for *BRAF* V600E mutant HCL. This treatment resulted in the development of chronic lymphocytic leukemia (CLL) from a preexisting *KRAS* G12D mutant monoclonal B-cell lymphocytosis (MBL). By next-generation sequencing of blood in an independent cohort of individuals with MBL and monoclonal gammopathy of undetermined significance (MGUS) we found that almost one third of these cases harbored clonal or subclonal activating *RAS* mutations. We conclude that patients with such RAS mutant precursor lesions have to be critically selected for and carefully monitored during tumor treatment with BRAFi.

## Methods

### Clinical Data

Informed consent was obtained from the reported patient with HCL and MBL/CLL as well as from eleven MBL and eleven MGUS control patients for the use of their diagnostic material as approved by the institutional review board (Ethikkommission der Ärztekammer Hamburg, Germany, project number PV4767).

### Isolation of Genomic DNA

Genomic DNA was isolated from peripheral blood mononuclear cells (PBMNCs) using GenElute Mammalian Genomic DNA Miniprep kit (Sigma-Aldrich, St. Louis, United States) according to the supplier’s instructions.

### Targeted Next-Generation Sequencing of Mutational Landscapes

Using a custom gene panel (QIAseq Targeted DNA Panel) gene regions of interest were amplified starting from 100 ng of PBMNC or bone marrow mononuclear cells (BMMNCs) genomic DNA, respectively. The panel covered the following genes: BRAF, HRAS, KRAS, and NRAS. Library preparation was performed according to the supplier’s instructions. Briefly, DNA was fragmented by a fragmentation enzyme mix and Illumina adapters containing 12-base unique molecular identifiers (UMIs) were ligated onto each DNA molecule. During target enrichment PCR, gene and hotspot specific primers amplify the regions of interest followed by a 21-cycle universal PCR, where all fragments of interest are amplified once more for final library construction. The final library was quantified by Qubit (Thermo Fisher Scientific Inc.) and fragment size was analyzed using an Agilent 2100 Bioanalyzer (Agilent technologies). Multiplex sequencing was performed with a 300-cycle dual indexed (8 nucleotides) paired-end run on a NextSeq sequencer (Illumina) at an estimated depth of 26 500 reads. Variant calling was performed using smCounter2 (13). The incorporated UMIs allow for elimination of biases that can be introduced during PCR amplification and therefore offer a highly accurate variant calling at a variant frequency level of ≥1%. For details of multiplex PCR and sequencing approach for the identification of RAS mutations in the MGUS patient cohort please refer to original publication (14).

### Next-Generation Sequencing (NGS) of Immunoglobulin Heavy Chain (IGH) Immune Repertoires and Data Analysis

For sensitive clonal monitoring, the *IGH* gene locus containing the rearranged *VH*, *DH* and *JH* segments was amplified by multiplex PCR from genomic DNA using previously published protocols (15). Amplicon extension with Illumina adapter sequences and unique indices was achieved through a second PCR reaction. Primers were purchased from Metabion (Martinsried, Germany) and PCRs were performed using Phusion HS II (Thermo Fisher Scientific Inc.) according to the supplier’s instructions. Finally, amplicons with the expected size were purified after agarose gel electrophoresis using the NucleoSpin^®^ Gel and PCR Clean-up kit (Macherey-Nagel). After amplicon quantification and quality control with a Qubit (Thermo Fisher Scientific Inc.) and an Agilent 2100 Bioanalyzer (Agilent technologies), respectively, sequencing was performed on an Illumina MiSeq platform (600–cycle single indexed, paired-end run). Analysis of the *IGH* locus was computed using the MiXCR analysis tool (16). Only productive sequences with a read count ≥2 were included in the analysis.

### Multicolor Flow Cytometry

Within 2 h of peripheral blood collection, erythrocyte lysis using a standard lysis buffer (ammonium chloride 8.29 g/l, EDTA 0.372 g/l, potassium hydrogen carbonate 1 g/l) was performed followed by flow cytometry using an 5-color flow cytometry panel established for routine clinical analysis of B cell disorders (all directly labeled antibodies purchased from Beckman Coulter). Measurements were performed on a FC500 cytometer (Beckman Coulter, Krefeld, Germany). B and CLL cell counts were calculated from absolute blood counts.

### Data Availability

Sequencing data generated for this study can be found in the European Nucleotide Archive (ENA). ID: PRJEB36480.

## Results

**FIGURE 1:**
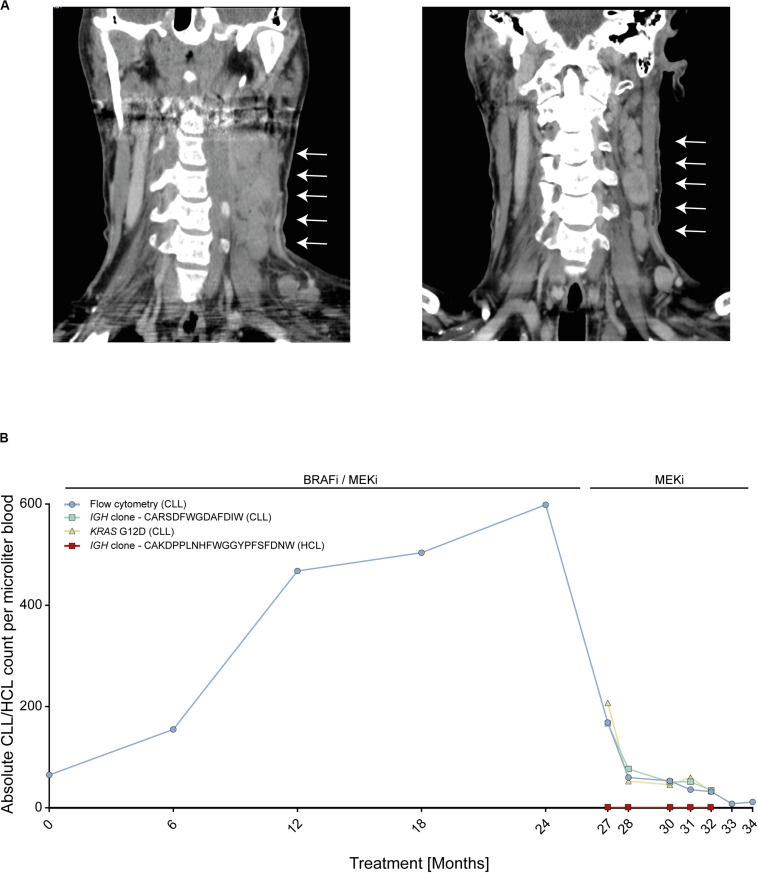
Patient with *BRAF* V600E-mutant hairy cell leukemia (HCL) and concomitant *RAS*-mutant B cell lymphocytosis (MBL) experiencing progression to chronic lymphocytic leukemia (CLL). **(A)** Computed tomography scans of cervical lymph nodes of patient with HCL and concomitant MBL/CLL. On the left, nodal progression on dual BRAFi/MEKi. On the right, nodal remission 3 months after BRAFi withdrawal. **(B)** Absolute MBL/CLL and HCL cell counts over time during combined BRAFi/MEKi and after BRAFi withdrawal. Cell counts were deduced from flow cytometry, *IGH* next-generation sequencing and *KRAS* G12D liquid biopsy analyses of peripheral blood. Clone CARSDFWGDAFDIW (green line) represents the MBL/CLL clone and clone CAKDPPLNHFWGGYPFSFDNW (red line) represents HCL clone. *IGH*, Immune globulin heavy locus. Arrows indicate cervical lymphnode.

We report on an instructive case of a patient with HCL and concomitant MBL, who received dual BRAFi/MEKi after his fifth HCL relapse. This patient responded quickly and achieved a durable HCL remission with excellent tolerability of both agents. Yet, 24 months after treatment initiation, he experienced transformation of MBL to overt CLL with accelerated nodal progression (Figure 1A). At this time, the HCL was in ongoing partial remission.

To address the molecular driver underlying the suspected BRAFi-induced accelerated progression, we performed next-generation sequencing using a gene panel including activating *RAS* mutations. We found that the initial MBL clone as well as the emerging CLL carried an activating *KRAS* G12D mutation, but no *BRAF* mutation, thus explaining the observed CLL progression. The *RAS* mutation was confirmed by Sanger sequencing from CLL tumor material (resected lymph node). Since enhanced MAPK signaling was expected to be attenuated upon withdrawal of the BRAFi, treatment was continued with the MEKi only, inducing a nodal CLL decline with maintained HCL control (Figure 1A). Despite the predominantly nodal CLL progression, multicolor flow cytometry and NGS-based blood monitoring of lymphoma clones over time (*IGH* and *KRAS*) provided a liquid biopsy window into disease dynamics showing a continuous increase of the MBL/CLL clone from the start of the dual BRAFi/MEKi treatment followed by rapid decline of the CLL clone upon withdrawal of the BRAFi (Figure 1B). MBL represents a common precursor lesion of CLL with relatively high population prevalence with some studies suggesting that up to 10% of individuals >40 years may harbor clonal B cell populations (17). Since activating *RAS* mutations may be present in lymphatic diseases and precursor states, we wished to explore *RAS* mutational frequencies in a small cohort of eleven MBL patients from our institution. To this end, we used an NGS targeted sequencing approach with an established sensitivity of ≥1%, since even subclonal *RAS* mutations in an MBL background are expected to be selected on therapeutic pressure with BRAFi and may give rise to transformation. This analysis showed that two of eleven (18%) MBL cases harbored activating *RAS* mutations (*HRAS* G13S and *KRAS* A146T). While the *HRAS* mutation was subclonal in MBL003 (present in 2.37% of MBL cells), the *KRAS* mutation in MBL005 was present in all MBL cells like in our index patient. Since a similar risk for transformation may exist in individuals with *RAS* mutant MGUS, a precursor lesion for multiple myeloma, we re-analyzed an NGS dataset from a cohort of eleven individuals with MGUS, that was previously published by our group, for the prevalence of *RAS* mutations (14). In this cohort, four out of eleven (36%) MGUS cases harbored activating *NRAS* mutations (NRAS G12C, G13 C/R/V, and Q61K). In two of these patients, more than one *NRAS* mutation was found in different subclones (Table 1).

**TABLE 1:** RAS mutations in individuals with MBL and MGUS.

ID number	RAS mutation
MBL 001	–
MBL 002	–
MBL 003	HRAS G13S
MBL 004	–
MBL 005	KRAS A146T
MBL 006	–
MBL 007	–
MBL 008	–
MBL 009	–
MBL 010	–
MBL 011	–
MGUS 002*	–
MGUS 003*	–
MGUS 005*	–
MGUS 006*	–
MGUS 007*	NRAS G13R, G13C, Q61K
MGUS 008*	–
MGUS 009*	NRAS G12C
MGUS 010*	NRAS G13V
MGUS 011*	–
MGUS 012*	NRAS G12C, Q61K
MGUS 013*	–

*From own previously published dataset (14).

## Discussion

Using a targeted drug that works on a specific molecular cancer lesion may appear a simple therapeutic concept. Yet, this concept must be considered oversimplified since systemic inhibition of oncogenic signaling not only operates on the tumor cell’s mutational landscape, but also on other (e.g., pre-malignant) tissue’s occult mutational landscapes. We currently face a growing body of evidence showing that BRAF inhibition (with and without concurrent MEK inhibition) may trigger the development of cancer from pre-neoplastic lesions (e.g., melanoma and non-melanoma skin cancer, pancreatic cancer) or drive progression of existing but unrecognized cancers (e.g., colon cancer) (5–12).

**FIGURE 2:**
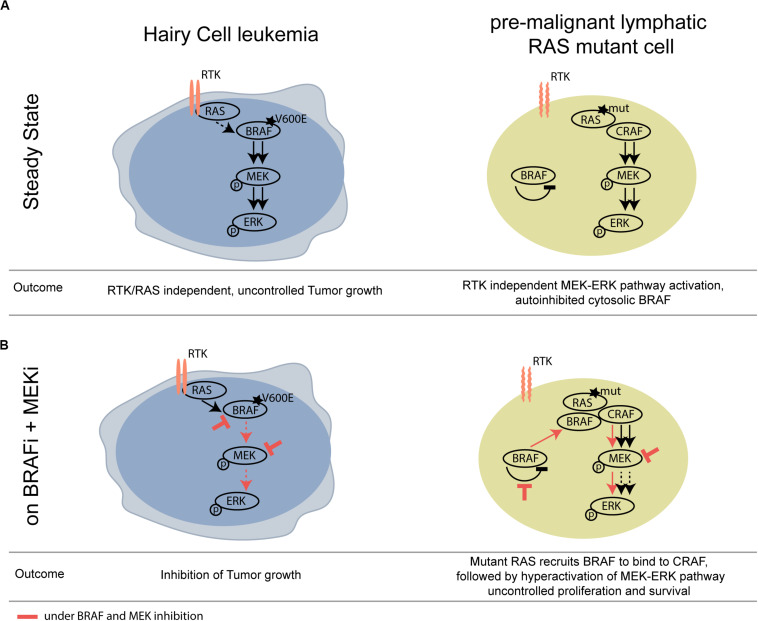
Schematic overview of differential activity of BRAFi/MEKi in *BRAF* V600E HCL versus *RAS* mutant pre-malignant lymphoid cells. **(A)** At steady state, mutant BRAF signals RTK/RAS independently and thereby drives tumor growth of HCL. **(B)** Upon BRAF and MEK inhibition, the activity of the MAPK pathway is strongly compromised in the HCL context, however, leading to paradoxical activation of the MAPK pathway in the *BRAF* wildtype, *RAS* mutant lymphoid clone. RTK, receptor tyrosine kinase.

We show that not only pre-cancerous skin lesions, but also hematological pre-cancerous lesions may progress upon BRAF inhibition. A mechanistic scheme illustrating this concept is shown in Figure 2.

In this case, we observed a steady increase of the MBL/CLL clone over time on BRAFi/MEKi treatment followed – after 2 years – by more important nodal progression. The rather slow dynamics over 2 years may be attributed by the concomitant MEKi, which, however, was not able to fully block paradoxical stimulation.

While in CLL, the overall frequency of *RAS* mutations appears to be below 10% (in low-resolution studies), in multiple myeloma and its precursor MGUS, deregulation of MAPK signaling via activating *RAS* mutations is a more common finding present on a clonal or subclonal level in 36–49% of patients depending on the resolution of the detection technology (14, 18). Our own previous data on a hospital cohort of non-hematooncological patients show that the overall frequencies of MBL and MGUS in individuals between 50 and over 80 years of age range from 0.9 up to 5% for MBL and 5.1 up to 11.8% for MGUS (19). Considering the *RAS* mutational frequencies of these precursor lesions (according to our data almost one third of cases with *RAS* mutations), this may result in significant numbers of patients at risk for the development of treatment-requiring hematological cancers on BRAF inhibition.

One of the limitations of our work consists in the fact that our data imply, but not directly proof that the *RAS* mutation is involved in paradoxical pathway activation in this case. The failure to directly show this is due to a lack of sufficient biological material from this case taken on BRAFi treatment. Nonetheless, the mechanistic concept of paradoxical BRAFi induced cancer progression in *RAS* mutant cells is well established and should therefore also apply to this case (3, 4, 20). Another limitation of our work is related to the fact that we don’t show any clinical case experiencing the development of myeloma from MGUS. Nevertheless, we chose to include *RAS* mutational analyzes for MGUS as well since this pre-malignant condition also concerns the B lineage and may result in similar problems. Clearly, these results at this point do not prove an increased risk for myeloma development in patients with *RAS* mutant MGUS on BRAFi, but in our view they should sound a note of caution.

Taken together, our study highlights several important points. First, caution must be exercised when using BRAFis in patients or populations that might harbor *RAS* mutant cells in skin, bowel, or other sites. Vigilant surveillance will remain critical in clinical protocols using these agents and even screening for such mutations (e.g., in circulating blood DNA) should be considered. Second, drug safety is likely to vary depending on the targeted molecule within a given signaling pathway, and although paradoxical activation of the MAPK pathway occurs with BRAF inhibition, the addition of a MEKi to a BRAFi might not be sufficient to overcome the paradoxical and unwanted ERK phosphorylation. Third, to prevent paradoxical stimulation in patients vitally requiring BRAFi treatment while harboring a pre-cancerous lesion with activating *RAS* mutation, a new generation of BRAFi termed “paradox breakers,” such as PLX8394 (Plexxikon^®^), is under development and will hopefully allow safe application of BRAFi in these vulnerable patients (21, 22).

Careful clinical monitoring and translational studies will be required to foster our understanding of safe application of BRAF inhibitors in routine clinical practice.

## Data Availability Statement

The datasets generated for this study can be found in the European Nucleotide Archive (ENA), ID: PRJEB36480.

## Ethics Statement

The studies involving human participants were reviewed and approved by the Ethikkommission der Ärztekammer Hamburg. The patients/participants provided their written informed consent to participate in this study.

## Author Contributions

AS and MB: conception and design. DS and MB: development of methodology. DS, HI, AS, MB, and CB: acquisition and interpretation of data, writing, review, and/or revision of the manuscript. All authors contributed to the article and approved the submitted version.

## Conflict of Interest

The authors declare that the research was conducted in the absence of any commercial or financial relationships that could be construed as a potential conflict of interest.
